# Triggers for freezing of gait in individuals with Parkinson’s disease: a systematic review

**DOI:** 10.3389/fneur.2023.1326300

**Published:** 2023-12-21

**Authors:** Carolina I. Conde, Charlotte Lang, Christian R. Baumann, Chris A. Easthope, William R. Taylor, Deepak K. Ravi

**Affiliations:** ^1^Institute for Biomechanics, ETH Zürich, Zürich, Switzerland; ^2^Department of Neurology, University Hospital Zurich, Zürich, Switzerland; ^3^The LOOP Zurich – Medical Research Center, Zürich, Switzerland; ^4^Lake Lucerne Institute, Vitznau, Switzerland; ^5^creneo Foundation – Center for Interdisciplinary Research, Vitznau, Switzerland

**Keywords:** Parkinson’s disease, freezing, gait, triggers, tasks

## Abstract

**Background:**

Freezing of Gait (FOG) is a motor symptom frequently observed in advanced Parkinson’s disease. However, due to its paroxysmal nature and diverse presentation, assessing FOG in a clinical setting can be challenging. Before FOG can be fully investigated, it is critical that a reliable experimental setting is established in which FOG can be evoked in a standardized manner, but the efficacy of various gait tasks and triggers for eliciting FOG remains unclear.

**Objectives:**

This study aimed to conduct a systematic review of the existing literature and evaluate the available evidence for the relationship between specific motor tasks, triggers, and FOG episodes in individuals with Parkinson’s disease (PwPD).

**Methods:**

We conducted a literature search on four online databases (PubMed, Web of Science, EMBASE, and Cochrane Library) using the keywords “Parkinson’s disease,” “Freezing of Gait”, “triggers” and “tasks”. A total of 128 articles met the inclusion criteria and were included in our analysis.

**Results:**

The review found that a wide range of gait tasks were employed in studies assessing FOG among PD patients. However, three tasks (turning, dual tasking, and straight walking) emerged as the most frequently used. Turning (28%) appears to be the most effective trigger for eliciting FOG in PwPD, followed by walking through a doorway (14%) and dual tasking (10%).

**Conclusion:**

This review thereby supports the utilisation of turning, especially a 360-degree turn, as a reliable trigger for FOG in PwPD. This finding could be beneficial to clinicians conducting clinical evaluations and researchers aiming to assess FOG in a laboratory environment.

## Introduction

Freezing of Gait (FOG) is a common disabling motor symptom that occurs in people with Parkinson’s disease (PwPD). In earlier stages of the disease, FOG is estimated to affect around 50% of PwPD, with the number increasing up to 80% as the disease progresses (1–3). FOG is defined as “a brief, episodic absence or marked reduction of forward progression of the feet despite the intention to walk” (4). In practice, it manifests itself in three different patterns: small ‘shuffling’ steps, alternating leg ‘trembling’ when stationary, and ‘akinesia’ with no discernible forward progression despite movement intention (4, 5). The severity and complexity of these patterns can vary significantly from patient to patient and can even fluctuate within the same individual at different times (6). FOG has a significant impact on mobility, frequently leads to falls, and reduces the quality of life for PwPD and their caregivers (7–10). Additionally, FOG can lead to increased anxiety and insecurity while walking (10–12).

Bardakan and colleagues describe three main pathophysiological models for FOG (13). The interference model suggests that FOG results from impaired crosstalk between cortical and subcortical areas, leading to over-inhibition of brainstem structures and reduced gait automaticity, especially during dual-task situations (14). Freezing episodes frequently observed in the OFF-medication state emphasize the significance of the dopaminergic pathway in relation to FOG (15–17). However, FOG also occurs in the ON medication state, suggesting that a hypodopaminergic state only partially explains FOG (18). Additionally, FOG commonly occurs when passing through doors or when approaching destinations (19). This could be explained by the perceptual dysfunction model, which describes a malfunction in visuomotor processing, resulting in an inability to adapt to dynamic environmental changes. Lastly, the executive dysfunction model accounts for FOG caused by obstacle avoidance. FOG could arise from a disconnection between the frontal lobe and the basal ganglia, as executive functions are called upon to compensate for the loss of automatic movement (13).

The normal control of gait requires coordinated excitation and inhibition of competing motor plans, which is compromised in people with FOG. FOG can occur during ongoing movements as a result of sudden temporal re-inhibition via the indirect pathway and a decrease in the disinhibition of the direct pathway. This leads to an over-inhibition of brainstem structures by the globus pallidus internus (GPi), hindering the initiation and execution of movements, consequently resulting in FOG. Overall, there is partial consensus that connectivity issues between the basal ganglia, the prefrontal cortex, and the frontoparietal areas as potential sources causing FOG (14, 15, 20). The presence of several hypotheses for the causes of FOG demonstrates the complexity of its pathophysiology (5).

Assessing FOG in a clinical setting can be challenging due to the paroxysmal nature and diverse presentation of symptoms (21–23). Multiple FOG-triggering settings have been associated with the condition, such as gait initiation (24, 25), turning (26–28), anxiety (11, 29), and walking in narrow spaces (30–32), among others. Three distinct phenomenological types of FOG have been identified in previous studies (5, 33): asymmetric-motor, sensory-attention, and anxious freezing. Asymmetric motor freezing occurs mainly during turning, movement initiation, or when walking through narrow passages (5, 33–35). Sensory attentive freezing is often a result of walking in the dark, walking through an unorganized space, or when the surface is sloped (5, 33). Proprioceptive disturbances such as in concomitant polyneuropathy can contribute to freezing or mask it. Lastly, anxious freezing is triggered in stressful situations, such as when under time pressure or dual tasking (5, 21, 29, 36).

Most FOG episodes occur outside of a clinical environment as the awareness of being observed (Hawthorne effect) may enhance walking performance (37), making it difficult to study the symptom during a doctor’s visit or in clinical studies (36, 38–42). Questionnaires such as the Movement Disorder Society – Unified Parkinson’s Disease Rating Scale (MDS-UPDRS) (43), the Freezing of Gait Questionnaire (FOG-Q) (44), or the New Freezing of Gait Questionnaire (NFOG-Q) (45) are available to confirm and determine the presence, frequency, and severity of FOG retrospectively (46). However, due to recall bias accompanied by a cognitive decline patients experience difficulties when self-assessing FOG based on their perception (47). Furthermore, minor differences in freezing severity may not be reliably detected by the NFOG-Q as it is a self-rated questionnaire and awareness of the freezing behaviour might change over time (47). As a result, videotaped gait analysis is currently the standard for FOG detection in clinics (15). During these analyses, patients often perform different gait tasks like turning or dual tasking, but there is a wide variation regarding the protocol and the tasks included in different studies and clinical examinations.

In order to better understand FOG and find effective therapies, many studies have attempted to recreate scenarios where FOG can be elicited in a reliable manner. Despite the availability of several questionnaires and analysis techniques to capture the appearance of freezing, the lack of coherent recommendations for reliably eliciting freezing episodes in both observational and interventional research has impeded progress in understanding FOG. This has led to sparse objective information regarding the detection and effectiveness of treatments in clinical practice (5, 14, 15, 34, 48). Some experimental studies have found certain walking tasks to be more effective in eliciting FOG, such as turning with a small radius or walking through doors (21, 49, 50). Based on these findings, some research groups have developed obstacle courses that combine various triggers, such as turns and narrow passages, as well as straight walking or dual tasking elements (51, 52). Additionally, virtual, or augmented reality technologies have been used to elicit FOG by providing individualized triggers and the ability to scale the difficulty and complexity of tasks (53–57). Although there is a large collection of gait tasks used to assess FOG, the effectiveness of these individual triggers in eliciting FOG remains elusive (29, 58, 59). Various studies, using various aforementioned triggers, were not able to elicit any freezing episodes during their protocol (19, 60, 61). As a first step to guide further development of triggering paradigms, now one of the challenges is to ascertain which motor task serves as the most efficient trigger for FOG.

Therefore, to address this challenge, the aim of this study was to conduct a systematic review of the existing literature to evaluate the evidence linking specific motor tasks and triggers to FOG episodes in PwPD. By gaining a comprehensive understanding of these triggers, we aim to lay the foundations for advancing current knowledge of the pathophysiological mechanisms underlying FOG, optimizing treatments, and enabling the development of new therapies.

## Methods

### Search strategy and selection criteria

**Figure 1:**
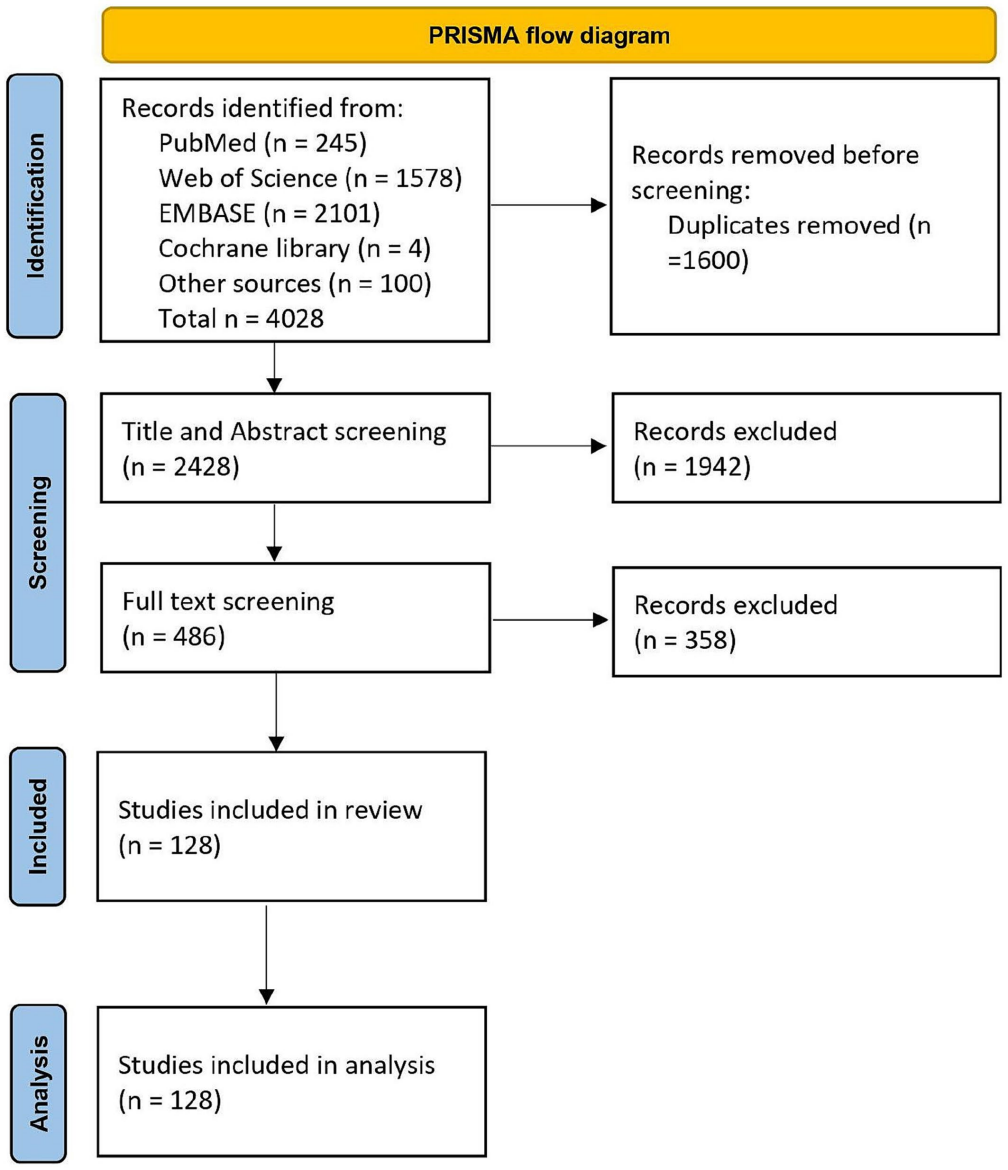
PRISMA flow diagram showing the studies included in the systematic review.

In October 2021, a literature search was carried out following the guidelines of the preferred reporting items for systematic reviews and meta-analysis (PRISMA) (62). Four online databases, namely PubMed, Web of Science, EMBASE, and Cochrane Library, were searched. In addition, articles that were identified prior to the commencement of the formal review were also included for screening (“other sources” in Figure 1). The search was conducted on articles published between 1998 and 2021 and was restricted to original articles published in English in a peer-reviewed journal. The complete search string is available as a Supplementary Methods 1. Furthermore, the complete review protocol has been registered in the PROSPERO database (CRD42022330511).

### Selection of studies and data extraction

The process of selecting studies was aided by the EPPI-Reviewer 4 (V.4.12.5.0, EPPI-Centre, UCL Institute of Education, University of London, London, UK) and EndNote (EndNote 20, Clarivate, Philadelphia, United States) software programs. Once duplicates were eliminated, two reviewers (CC, DKR) independently screened the titles, abstracts, and full texts, resolving any discrepancies through consensus. Studies were considered eligible if they included adult patients with PD and reported on FOG during a walking task. Studies were excluded if they reported FOG outcomes during stepping or turning in place tasks, gait initiation, unsupervised daily-life environments, or if participants walked using walking aids.

A spreadsheet (MS Excel, version 2018, Microsoft Corporation, Washington, United States) was used by one reviewer (CC) to extract the following information:

- Publication details such as author names, title, and publication year
- Study information including the number of individuals in freezing and non-freezing, gait tasks performed, and the technology / assessment scores used for FOG assessment
- Participant demographics such as age, gender, disease duration, disease stage, medication, and clinical assessments [MDS-UPDRS score (51) and Hoehn and Yahr score (60)]
- FOG outcomes such as total FOG count, participants with FOG, percentage of time spent frozen, trigger, gait task, etc.

If any information was missing or needed clarification, the authors of the included studies were contacted for additional details.

### Data synthesis

In order to assess the most effective triggers, the data of all studies that stated the gait task triggering FOG were extracted and normalized by the number of studies reporting the same trigger. For every activity, the numbers of FOG episodes (“Total FOG count”, Supplementary Table S1) were summed and normalized by the number of studies reporting FOG episodes triggered by the respective task. Multiple studies conducted measurements while participants were both ON and OFF medication states or involved different participant groups, such as freezers and non-freezers, among others (“Cohort”, Supplementary Table S1). In such cases, reported outcomes, such as the number of FOG episodes for all states and groups, were extracted and utilized for the analysis. Plots were created in the RStudio Software (R version 4.1.3, R Core Team, Vienna, Austria) using the ggplot2 package (version 3.4.2) (63).

## Results

### Study selection and characteristics

After an initial search that yielded almost 4,000 articles, 1,600 were identified as duplicates. Following a screening of titles and abstracts, 486 articles remained eligible, and 128 of them met the inclusion criteria (Figure 1).

Among the 128 studies included, 96 reported either the number or the percentage of participants who experienced FOG during the gait trials. In total, these studies included 2,804 patients, of whom 2,156 (77%) were categorized as ‘freezers’, either based on clinical assessments conducted through physical examinations or through self-reporting. In 116 out of the 128 studies included, 11,599 FOG episodes were reported. The mean age of all PD participants was 66.9 years, ranging from 51.0 to 76.8 years. Furthermore, the average disease severity was determined based on Hoehn and Yahr scale, was available in 81 out of the 128 studies, resulting in an average disease stage of 2.5, indicative of mild bilateral disease (64). Overall, more male participants (64%) were included in the studies compared to female participants (36%). Two studies reported only the median value for FOG outcomes (average FOG count, FOG duration and percentage time with FOG). For the analysis in this study, the median value from both studies was utilized as the mean value due to sufficient sample sizes in both studies.

#### Patients with FOG

**Figure 2:**
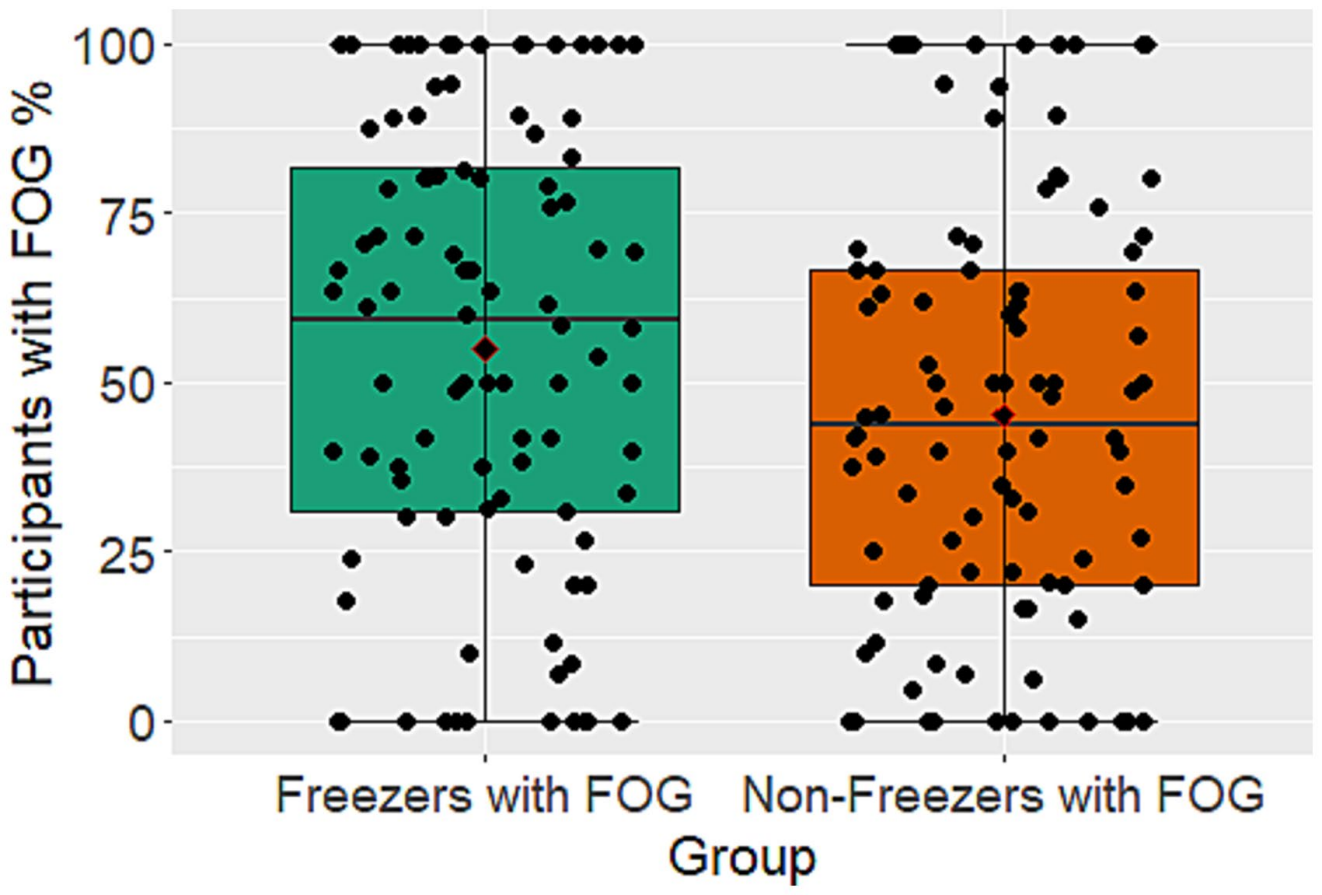
Boxplots showing the percentage of participants with FOG during gait trials for PwPD that are not known as freezers and the known freezers subgroup. Illustrated is the median with 25% and 75% confidence intervals.

The patient cohort sample size, as well as the percentage of participants who experienced FOG, varied greatly between studies from 4 to 305 subjects and 0% to 100%, respectively (Figure 2). Looking at all participants with PD, 12 studies were not able to elicit any FOG in their test population, while 9 studies triggered FOG in all their participants. A total of 316 participants (average 26 participants per study) belongs to the group of studies with no FOG while the number of participants in studies with 100% FOG sums up to 121 (average 13). Out of the previously mentioned 80% of participants that were categorized as freezers, 54% experienced freezing in the course of the gait experiment.

#### Tasks

**Figure 3:**
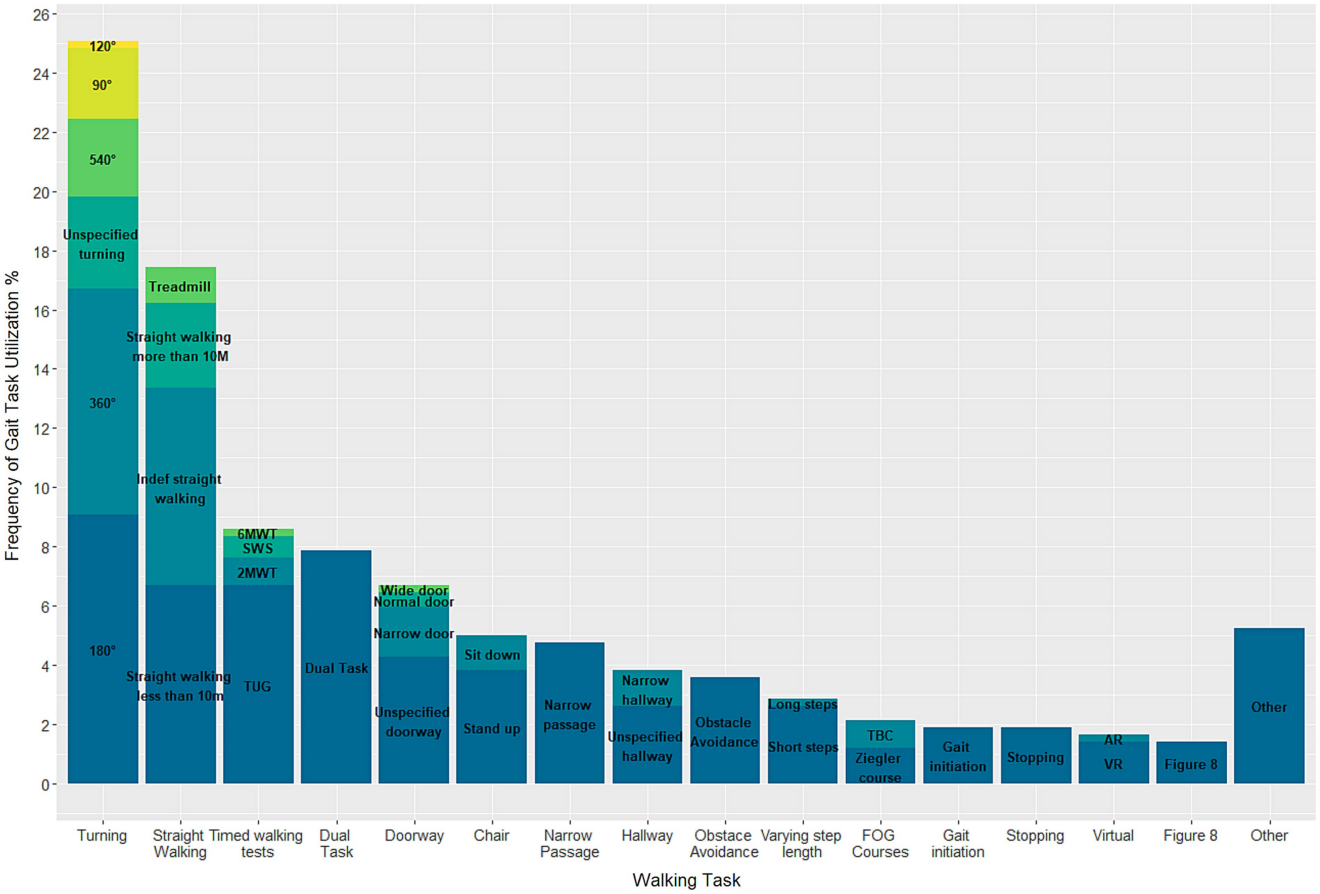
Stacked barplot displaying the different gait tasks used.

In total, 40 different gait tasks were reported and grouped into 16 categories based on higher-level tasks. Of the 128 included articles, only 22 studies exposed their participants to a single walking task, while the remainder requested their participants to perform at least two tasks. Turning of 180° (*n* = 38 studies) was the most used task, followed by 360° turning (*n* = 32 studies) (Figure 3). A few tasks were only conducted in a singular study, namely: long steps, augmented reality, turning of 120°, passing a wide door, and a *Six Minute Walking Test*. For the Turning and Barrier Course (TBC) subjects were “instructed to stand up, walk around the dividers twice in an ellipse, and then walk in a ‘figure eight’, around and through the opening between the dividers, twice, before sitting down again” (65). Execution of the Ziegler course included a “Stand Up and Go test crossing through a doorway and then, turning back” (66). Details on the study designs and execution of the other gait tasks can be found in the Supplementary material (Supplementary Table S1).

#### Trigger

**Figure 4:**
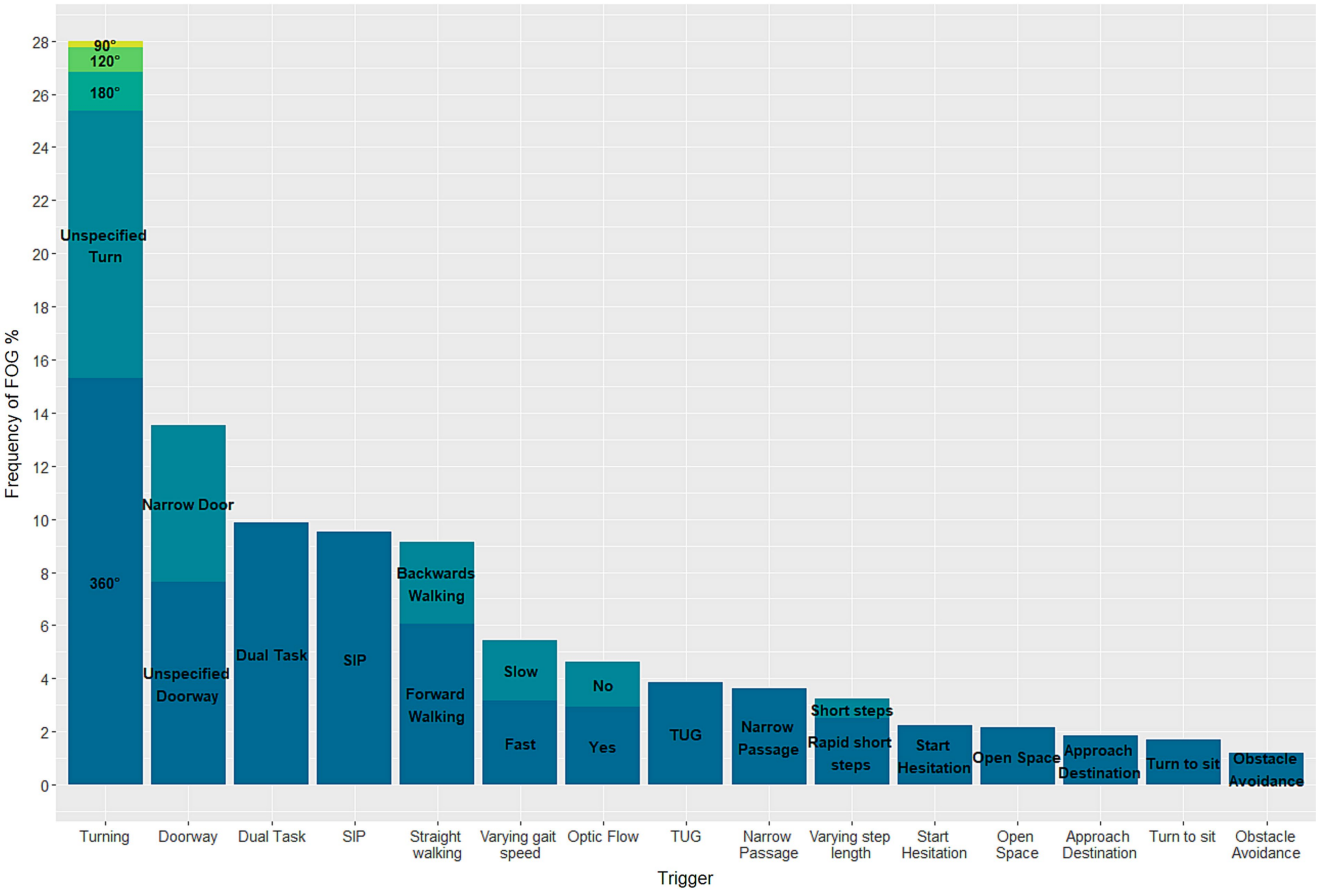
Stacked barplot demonstrating the triggers eliciting FOG in known freezers during gait trials.

Among the included studies, 26 specified the gait tasks that triggered FOG. This includes studies that examine participants in both ON- and OFF-medication condition. In total 24 different triggers were identified. Of these, the most effective trigger was 360° turning, which was responsible for 15.3% of FOG episodes recorded in the aforementioned studies (Figure 4). In addition, unspecified turning (10.1%), dual tasking (9.9%), stepping in place (9.5%), and passing a doorway (7.6%) were all activities with relatively high rates of eliciting FOG.

## Discussion

Freezing of Gait is a debilitating symptom experienced by many people with Parkinson’s disease. However, due to challenges in eliciting FOG in controlled laboratory or clinical settings, there has been a relative lack of research focused specifically on this phenomenon. Here, the challenge was to identify gait tasks most effective at triggering FOG. To address this, a systematic review of the literature was conducted, aiming to provide valuable insights into the tasks most likely to elicit FOG. The review revealed that turning is not only the most frequently studied gait task but also the most effective gait task for inducing FOG in PwPD (28%), followed by passing a doorway and dual tasking. This effort supports researchers in studying the underlying mechanisms of the symptom and developing effective interventions to improve the quality of life of PwPD.

Several theories exist surrounding the potent induction of FOG by turning. One hypothesis posited by previous studies suggests that FOG is caused by a delay in maximum head-pelvis separation, resulting in inadequate preparation for directional changes (27, 59). Moreover, the asymmetrical stepping pattern induced by turning or the reduced ability of PwPD to adapt to a new gait pattern may also contribute to this phenomenon (67).

Cowie et al. (49) reported a study that observed a slowing of walking in PwPD when approaching doorways, which they attributed to impaired visuomotor processing. However, an alternative explanation could be that attention is diverted from walking when approaching a doorway, leading to reliance on automatic movement, which can be disrupted in some PwPD (68, 69). Previous studies have demonstrated that dual tasking can lead to increased gait arrhythmicity and unsteadiness, as well as reduced step length and walking speed in PwPD (61, 70, 71). This suggests that dual tasking may contribute to the occurrence of FOG, as patients divert their attention away from the gait task towards the secondary task, thereby increasing cognitive demand. Although several other gait tasks elicited FOG to a lesser extent, the diversity of triggers suggests that various brain areas and mechanisms are involved in the occurrence of FOG. Human movement involves a complex interplay of several brain areas (14, 72, 73), and any damage along the neural chain could therefore influence movement in different ways (Table 1) (14).

**Table 1:** Gait and possible neural mechanisms of different gait tasks.

Task	Possible movement deficit	Possible neural deficit
Turning	Re-orientation to a new direction (74). Smaller step length on inner side of turning cycle than outside (26).	The basal ganglia provides phasic cues to the supplementary motor area (SMA), which regulates the bimanual coordination of movements (75). The striatum is activated contralateral to the intended direction (76).
Dual task	Execution of a primary task (main focus of attention) while performing a secondary task simultaneously (77).	Cognitive prioritization of the executed tasks, the more attention-demanding task is controlled by frontal cortical areas (78). The processing of the more automatic task (decreased dependency of attention) is shifted to the basal ganglia-circuit with input to the brainstem (69, 77).
Obstacles doorways, narrow passages	Adjustment of ongoing locomotor patterns in order to adapt to changes in the environment (13).	Visual information in the visual cortex are translated to visuospatial information in the posterior parietal cortex (PPC) and motor output is generated by projections of the PPC to frontal motor regions. The visuospatial input and the motor output are compared online. The integration of proprioceptive and motor information might involve the basal ganglia (79).

In this systematic review, it was found that three gait tasks, namely turning, dual tasking, and straight walking, were performed in at least half of the 128 studies analysed. Other gait tasks were performed much less frequently. The reasons for this preference for certain gait tasks can only be speculated on. It is possible that the choice of tasks was influenced by previous studies reporting successful triggering with a specific task or the ease of preparation and infrastructure required for the task. For instance, tests such as the TUG can be performed with minimal preparation, and adding a dual task to a pre-existing gait task can also be relatively simple.

A wide variation was observed in the percentage of participants experiencing FOG during gait assessment, ranging from 0 to 100% for both known freezers and PwPD (Figure 4). This variability is likely due to differences in study protocols, number of walking trials, medication status, and disease severity. However, the paroxysmal and unpredictable nature of FOG, influenced by environmental, emotional, and cognitive factors, is also a contributing factor (4, 21). In the studies with no FOG episodes the most commonly examined task was straight walking. Nonetheless, all of the studies encompassed a diverse range of additional tasks, rendering it unfeasible to reach any definitive conclusions. In comparison to a previous study of almost a thousand participants, which reported a FOG prevalence of around one-third (80), the current study, involving a larger population of over 95 studies with exposure to a variety of tasks, showed a higher prevalence of around 50% for both PwPD and the subgroup of only Freezers (76). This could enable a more enhanced exploration of the underlying mechanisms by the use of more effective triggers.

The male predominance in our sample is consistent with the fact that males are twice as likely to be affected by the disease than women (81). The broad range of disease severity from I to almost IV further complicates comparison of study outcomes. Despite these limitations, this study provides a valuable contribution to the literature on FOG in PwPD and highlights the need for further investigation and standardization of FOG assessment protocols. With the study’s broad inclusion criteria, this is the first survey to comprehensively examine activities that elicit FOG by reviewing a large number of studies. However, the diverse nature of the studies included with varying execution of the conducted gait tasks posed a challenge in comparing outcomes. Additionally, a limitation of the study is the absence of a bias assessment to evaluate the quality of the included studies. Nonetheless, the study’s strengths, such as its comprehensive review and broad inclusion criteria, outweigh its limitations and contribute significantly to the current understanding of FOG in PwPD.

A noteworthy discovery from this study was the relatively low number of studies that focused on the triggers of FOG. The reason for this may be attributed to the challenge of accurately determining the cause of FOG episodes, which can be influenced by various emotional, environmental, and other factors. Interestingly, the frequency of FOG episodes did not significantly differ between the Freezers group and the entire PD patient population. This could be due to the unpredictable nature of FOG (10), or even the feeling of being observed and therefore increased attention while undertaking functional assessments in clinical and laboratory settings (34).

In order to systematically study the underlying mechanisms of FOG, it is important to know which gait tasks act as the most efficient triggers for FOG. Current research is moving increasingly towards real-life assessment using IMUs (82). However, this approach only informs about behavioural changes and does not provide information about the specific triggers that cause FOG. Therefore, more studies that focus specifically on the triggers of FOG are needed. Based on the analysis of 26 studies that specified the gait tasks triggering FOG, this review provides evidence suggesting that turning is currently the most prominent trigger of FOG. Nevertheless, several other gait tasks also possess the capability to elicit FOG to some extent. Based on these findings, turning might be the most effective task for FOG assessment in clinical examinations, but future studies should continue to cover a broad range of potential triggers in their protocol, as this can facilitate inference from phenomenological observation to underlying mechanisms. Using different gait tasks separately according to a standardized protocol could simplify the process of precisely identifying and distinguishing the exact triggers of a FOG episode. However, the identification and categorization of triggers for FOG can be a complex task due to their subjective nature. Importantly, researchers should systematically annotate the cause of the FOG episode. This is particularly noteworthy as, until now, only a minority of studies explicitly report the actual triggers of FOG.

As the population continues to age, the prevalence of neurodegenerative diseases like PD is expected to rise. Thus, it is crucial to fully comprehend the symptoms, diagnosis, and treatment options associated with this condition. However, despite significant advancements in this area, research on the occurrence, severity, epidemiology, and underlying causes of FOG remains scarce. Therefore, this systematic review’s findings on triggers for FOG in PwPD will be valuable in guiding future research and clinical applications, aiding in the selection of assessments for FOG. Emerging technologies such as augmented or virtual reality hold promise in this field, as they can be utilized to evaluate, diagnose, and cue FOG effectively. These technologies can incorporate individual triggers as building blocks, allowing for the creation of personalized walking courses tailored to an individual’s preferred difficulty level.

## Conclusion

This review offers a significant contribution to the understanding of FOG triggers in PwPD by providing a comprehensive overview. The results indicate that turning is the most effective trigger for FOG in PwPD, followed by walking through a doorway and dual tasking. These findings have potential applications for researchers designing studies on FOG, clinicians evaluating patients with PD, and the development of interventions to manage or prevent FOG. Implementing the results to design coherent recommendations in research as well as clinical evaluation can lead to a better understanding of FOG and therefore an improvement of current treatment.

## Author contributions

CC: Data curation, Formal analysis, Investigation, Visualization, Writing – original draft, Writing – review & editing, Methodology. CL: Formal analysis, Visualization, Writing – review & editing. CB: Writing – review & editing. CE: Writing – review & editing. WT: Funding acquisition, Resources, Writing – review & editing. DR: Conceptualization, Data curation, Formal analysis, Funding acquisition, Investigation, Methodology, Project administration, Resources, Supervision, Writing – original draft, Writing – review & editing.
